# The Caribbean American Dementia and Aging Study: protocol for a population-based study of older adult health and dementia in Cuba, the Dominican Republic, and Puerto Rico

**DOI:** 10.1186/s12877-025-06131-0

**Published:** 2025-08-29

**Authors:** Mao-Mei Liu, Jorge Llibre-Guerra, Chris Soria, Jing Li, Tania Zayas Llerena, Guillermina Rodriguez, Daisy Acosta, Ivonne Jiménez Velázquez, Juan J. Llibre-Rodriguez, William H. Dow

**Affiliations:** 1 Department of Demography, University of California at Berkeley, Berkeley, USA; 2 Department of Neurology, School of Medicine, Washington University in St. Louis, St. Louis, USA; 3 Department of Pharmacy, University of Washington, Seattle, USA; 4 Dementia Research Unit, Medical University of Havana, Havana, Cuba; 5 Universidad Nacional Pedro Henríquez Ureña (UNPHU), Santo Domingo, Dominican Republic; 6 School of Medicine, University of Puerto Rico, San Juan, Puerto Rico; 7 School of Public Health, University of California at Berkeley, Berkeley, USA

**Keywords:** Aging, Population health, Population-based studies, Dementia, Alzheimer’s disease, International comparison, Caribbean, Puerto Rico, Dominican Republic, Cuba

## Abstract

**Background:**

The Hispanic Caribbean region is rapidly aging but national population-based aging surveys are rare. The Caribbean American Dementia and Aging Study (CADAS) is a multi-purpose household study of aging with a particular focus on the life course determinants and consequences of health and dementia in three countries with many similarities but divergent recent histories: Puerto Rico, Dominican Republic, and Cuba.

**Methods:**

CADAS will survey population-based samples of adults ages 65 and over. In Puerto Rico and Dominican Republic these will be nationally representative samples of 1,500 adults each; the Cuban survey will include 1,500 adults from purposively selected urban and rural areas. We are collecting detailed data modules that encompass individual health metrics, sociodemographic background, physical and neurological exam, cognitive function, and household economic status. Data modules are generally harmonized with related Health and Retirement Surveys (HRS) around the world. Participant cognitive assessment protocols and informant reports were designed to be broadly harmonized with the 10/66 Dementia Research Group assessments and the Harmonized Cognitive Assessment Protocol (HCAP). Data collection is in progress in all three countries. For a subset of CADAS data, clinicians will provide online clinical consensus diagnoses based on National Institute on Aging and Alzheimer’s Association (NIA-AA) ratings.

**Discussion:**

With this study, we will contribute nationally representative data on health, dementia and cognitive aging in Dominican Republic and Puerto Rico, as well as harmonized data in Cuba to expand understanding of aging and dementia in the Hispanic Caribbean and promote cross-national comparative research around the world.

## Background

As populations worldwide age, understanding the implications for families and societies becomes crucial, and robust cross-national scientific research is necessary to shape policy dialogue and decisions. The bulk of aging research continues to focus on high-income countries, but aging research is particularly needed among low- and middle-income countries, where the dynamics of aging may differ significantly. One of the developing world’s regions that is most rapidly aging is Latin America and the Caribbean [1]. The Spanish-speaking Caribbean (Cuba, Dominican Republic, and Puerto Rico) is of special interest given its rapidly aging populations, driven by long-term patterns of low fertility and reduced mortality [2] and recent high levels of outmigration by working age adults [3] which augment large existing migrant and migrant-descendent communities in the mainland United States [4]. Their connected histories, richly varying societal structures and population diversity together offer great promise for better understanding of health and aging.

As a key cause of disability and dependency among older adults, Alzheimer’s disease and related dementias (ADRD) represent a challenge to societies worldwide [5] and to Latin America and the Caribbean in particular [6]. While ADRD is neither normal aging nor the exclusive domain of older adults, age remains the most powerful risk factor for dementia. Existing studies from the 10/66 Dementia Research Group find a dementia prevalence among metro-dwelling older adults (aged 65+) of 10–12% in the Spanish-speaking Caribbean: Cuba, the Dominican Republic, and Puerto Rico [7]. To date, however, no nationwide epidemiological studies exist of dementia in any of these countries.

The 10/66 study was the ground-breaking population-based effort to study dementia among older adults in low and middle-income countries and led the major previous data collection efforts in the Spanish-speaking Caribbean, using innovative instruments to collect comparative data to estimate dementia prevalence and risk factors [8, 9]. The 10/66 collected in-depth cognitive and neuropsychological testing, geriatric assessment, physical exam, socio-demographic background of participants, as well as reports by key informants and information about the participants’ households. In the Caribbean, the 10/66 group focused on study sites in metropolitan regions of Havana City and Matanzas in Cuba, Santo Domingo in Dominican Republic and San Juan in Puerto Rico, but did not include samples from rural or other areas.

Building upon previous U.S.-based studies and the work of the 10/66 Dementia Research Group, the Harmonized Cognitive Assessment Protocol (HCAP) – a common set of cognitive and neuropsychological tests and informant reports – was designed to assess dementia and mild cognitive impairment in the U.S. Health and Retirement Survey and its growing network of international sister studies [10]. The use of HCAP in the U.S. and beyond will increase opportunities for comparative research on dementia’s prevalence, risk factors and consequences. To date, in addition to the U.S., the HCAP has been implemented successfully in Europe, e.g. the English Longitudinal Study of Ageing (ELSA) [11], the Irish Longitudinal Study on Ageing (TILDA) [12], the Northern Ireland Cohort for the Longitudinal Study of Ageing (NICOLA) [13], and the Survey of Health, Aging and Retirement in Europe (SHARE) [14]; in Asia, e.g. the China Health and Retirement Longitudinal Study (CHARLS) [15] and the Longitudinal Aging Study in India (LASI) – Diagnostic Assessment of Dementia (LASI-DAD) [16]; in Africa, e.g. South Africa’s Health and Aging in Africa Longitudinal Study of an INDEPTH Community (HAALSI) Dementia study [17], Kenya’s Longitudinal Study of Health and Ageing in Kenya (LOSHAK) [18] and the Kenya Life Panel Study; and in Latin America, e.g. the Mexican Health and Aging Study (MHAS) – Cognitive Aging Ancillary Study (Mex-Cog) [19] and the Chile Cognitive Aging Study (Chile-Cog) [20]. These studies have all implemented HCAP in the later waves of ongoing longitudinal studies; CADAS differs in that the HCAP cognitive assessments are being collected during the baseline study wave, along with an extensive set of other measures, thus survey design tradeoffs have been made to ensure that respondent burden is manageable.

In the Spanish-speaking Caribbean, the National Institute on Aging (grant R01AG064778) has funded one wave of Caribbean American Dementia and Aging Study’s (CADAS) HCAP data collection in the Dominican Republic and Puerto Rico, while the CADAS sister study in Cuba is self-funded. CADAS in Puerto Rico and the Dominican Republic, and the sister study in Cuba thereby contributes population-based samples in all three countries, including nationally representative samples in the Dominican Republic and Puerto Rico. CADAS is designed to harmonize with both the earlier 10/66 cognitive surveys and the growing family of HCAP surveys, to allow for extensive cross-national comparative research with past and future studies. Longitudinal waves of CADAS data collection are not yet confirmed, and will depend on future funding availability.

## Methods/design

### Study aims

CADAS aims to enhance research on aging and dementia within these Spanish-speaking Caribbean populations by collecting and analyzing high-quality data on older adult aging, cognitive health, dementia, and associated risk factors. The CADAS project team seeks to:

1. Collect data on older adult health and cognition in nationally-representative samples of Puerto Rico (*n* = 1,500) and the Dominican Republic (*n* = 1,500) and in population-based samples of selected areas of Cuba (*n* = 1,500).
2. Collect and store blood specimens in Puerto Rico and Dominican Republic (*n* = 3,000).
3. Establish online clinical consensus diagnoses (*n* = 900).
4. Estimate the prevalence of dementia in each country.
5. Examine how aging and dementia are associated with hypothesized life course socio-economic determinants.
6. Investigate the costs of aging and dementia on families and societies.
7. Make publicly available to researchers de-identified data from Puerto Rico and the Dominican Republic. Data from Cuba will require an application for use and the approval of the principal investigator in Cuba.

### Sample

CADAS is a baseline study of older adults aged 65 and above in the Spanish-speaking Caribbean and collects nationally representative samples in the Dominican Republic and Puerto Rico; in Cuba, respondents are drawn from a purposive set of urban and rural primary sampling units (PSUs). The target sample is 4,500 older adults (1,500 from each country). In both Puerto Rico and Dominican Republic, CADAS utilized their Censuses (from 2020 to 2010, respectively) as a sampling frame for drawing PSUs; in each, approximately 80 self-weighted PSUs were randomly selected. The Cuban sample includes PSUs in both urban and rural areas, but due to field conditions (a fuel crisis that precluded nationwide fieldwork travel), these were selected purposively in the west of the country. We plan to provide Census-based sampling weights to allow researchers to generalize estimates to the relevant Cuban population. Interviews were attempted among up to three age-eligible respondents in all households in the PSU with residents ages 65 and over. CADAS will construct sample weights correcting for non-response to allow researchers to make representative inferences to the reference population of adults aged 65 and older.

### Recruitment strategy

CADAS data collection proceeds in multiple stages. In Puerto Rico and Dominican Republic, a door-knocking team first canvasses the PSU, conducting a census of households to identify all age-eligible participants and count non-eligible household members. In Cuba, age-eligibles are identified from the comprehensive primary care clinic rosters of all residents in the PSU. In households with more than three age-eligibles, a Kish procedure is used to select the three interviewees to be recruited. Next, our trained interviewers visit the households to provide study details, invite study participation, and to attain signed informed consent; for target interviewees with cognitive impairment, a legal representative must provide signed informed consent. Participants complete a series of cognitive assessments, interviews about themselves and their households, and a physical assessment; a household/family member or neighbor separately completes an “informant” interview about the participant’s cognitive status. These interviews typically require about three hours in the household. Subsequently, in Puerto Rico and Dominican Republic consenting participants have their blood drawn (36 ml) by a phlebotomist during a separate scheduled visit (ideally within 30 days from the initial interview).

All CADAS data collection teams receive extensive training conducted by the same expert clinicians and experienced project staff at each site. This training includes: the overall CADAS study, door-knocking and how to approach eligible households and participants, informed consent, technical and practical training on how to administer cognitive assessments, informant interviews, physical exams, socio-demographic interviews, household questionnaire, technical training on how to use tablets for data collection, data upload and quality assurance, etc. Following the training session, interviewers were required to complete cognitive assessments with mock participants, which were reviewed for quality control review and correction as needed. Only upon passing this quality control review were the interviewers certified to commence fieldwork. During the period of data collection, all teams are closely supervised by country project coordinators and the local principal investigator.

CADAS data collection started in June 2023 in Cuba and the Dominican Republic and February 2024 in Puerto Rico and is expected to be complete by late 2025.

### Informed consent

The CADAS informed consent process involves: 1) participant’s consent to participate in the cognitive testing (including audio recording of certain tests for validation/scoring), health assessment, socio-demographic survey, and blood collection; 2) informant’s consent to complete an informant interview. Interview teams collect informed consent directly from participants. If a participant is cognitively impaired, we seek consent from a close family member (*e.g.* spouse, adult child) who can serve as legal proxy. The interviewer will read the consent forms to participants who cannot read or navigate them easily. Participants, their proxies and/or informants are free to halt participation at any time.

### Data elements

CADAS is a multi-national interdisciplinary study of aging (physical, cognitive and mental health, activities, functional state, etc.) that seeks to characterize demographic, behavioral, occupational, socio-economic, household and biological factors of aging. To collect these data, the CADAS study protocol includes a detailed set of socio-demographic and household questionnaires, physical exam, interviewer observation of the neighborhood, cognitive assessments, informant interview, and (in Puerto Rico and Dominican Republic) venous blood collection. While data harmonization is the priority, we have included different options or language adaptation for each country as needed. These adaptations include details such as birth region, healthcare and social insurance regimes, and minor differences in Spanish colloquialisms. All incomes and expenditures are recorded in each country’s currency.

#### Sociodemographic and health data

CADAS collects comprehensive data about participant’s socio-demographic background, household and neighborhood. The socio-demographic questionnaire includes information about participants’ early life conditions; parents, siblings and family support; current and past socioeconomic situation; international migrations; current social environment and well-being; current and past work; current and past health; sleep and stress/anxiety; pain; activities of daily living; health behaviors; access and use of health services. The household questionnaire includes detailed data about household housing conditions, assets, debts and income sources, and the neighborhood questionnaire collects observations about the participant’s neighborhood (e.g., amenities, walkability, noise, etc.). CADAS in Puerto Rico and the Dominican Republic also collects geographic coordinates, for merging exposome data such as NO2, PM2.5, light at night, temperature, precipitation, etc. (funded by a subaward from NIH grant 2R01AG030153-17S1 at the University of Southern California under principal investigator Jinkook Lee). Table 1 documents the kinds of socio-demographic data collected during CADAS household interviews, individual interviews, physical exams, and neighborhood observations.

CADAS conducts a comprehensive physical exam that takes about 20 minutes. As in many sister studies, specially trained CADAS staff measure participants’ blood pressure, hand grip strength, waist circumference, standing height, knee height, weight, etc. Less common in HCAP sister studies is CADAS’ additional structured neurological examination to assess various aspects of neurological function; our protocol is drawn largely from the 10/66 neurological exam protocol. This includes cranial nerve assessment (eye movements), motor system evaluation (muscle tone, limb strength, tremors, pronator drift, tendon reflexes), coordination and gait analysis (coordination, dysdiadochokinesis, reciprocal coordination, gait), and reflexes and frontal lobe function (primitive reflexes, Luria’s Test).

**Table 1:**
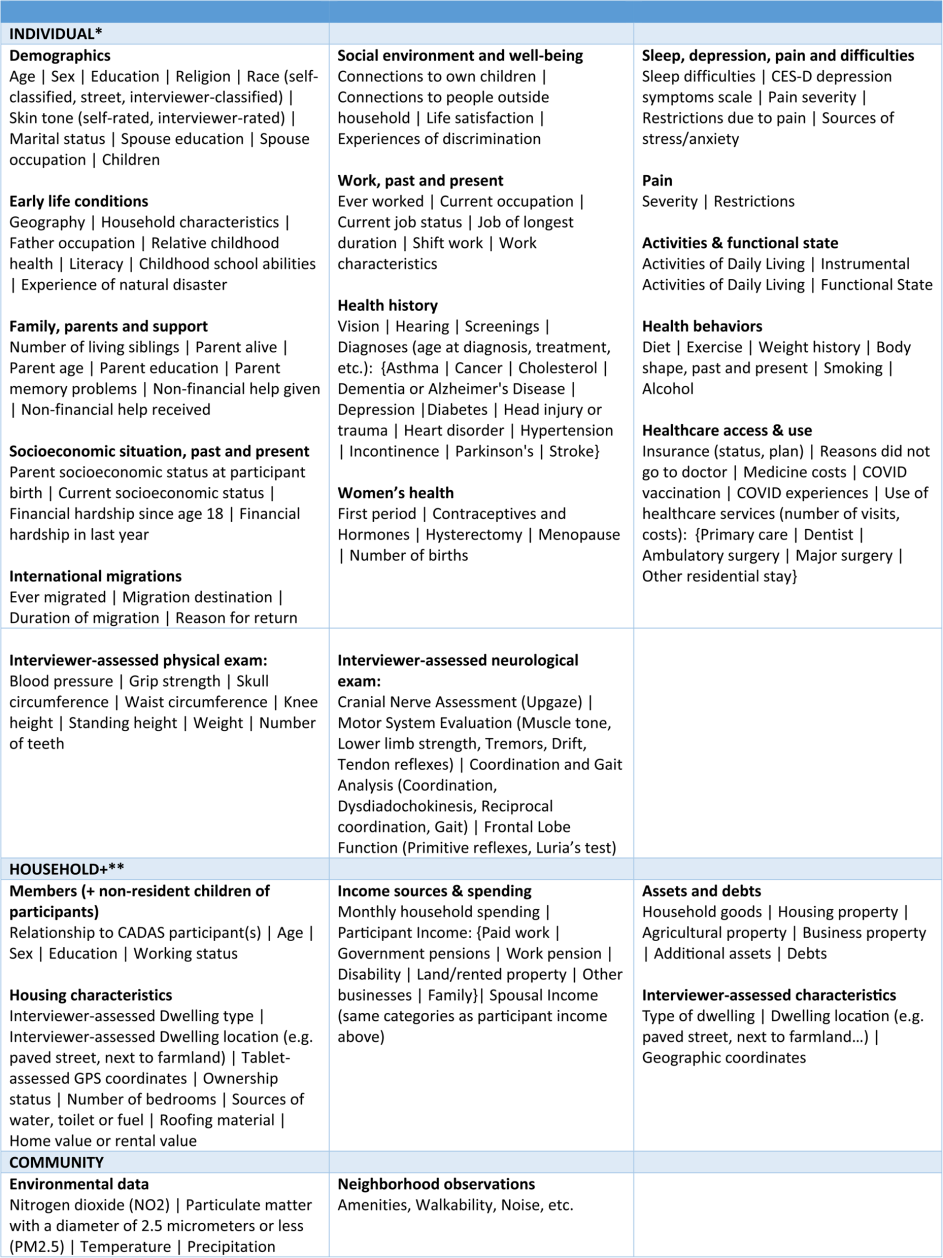
Sociodemographic, health and community data collected during CADAS interviews

*Individual data is self-reported unless otherwise noted

**Household data reported by household member unless otherwise noted

#### Cognitive assessment

CADAS has a particular focus on cognitive functioning and dementia and designed its cognitive test protocol based on the Harmonized Cognitive Assessment Protocol (HCAP), but with attention also to backwards harmonizing to the earlier 10/66 studies in these countries and elsewhere. CADAS conducted extensive pretesting, piloting and adaptation in order to maximize harmonization with other HCAP [21] and 10/66 [8, 9] families of studies, while ensuring quality data collection. The tests include word recall, semantic fluency, symbol cancellation [23], timed backward counting [24], Community Screening Instrument for Dementia (CSI-D) [25], logical memory, Telephone Interview for Cognitive Status (TICS) [26] and circle, rhombus, overlapping rectangles, cube drawing [27]. Table 2 presents the set of cognitive tests included in CADAS, compared to those administered in 10/66 and HCAP.

**Table 2:** CADAS Cognitive Test Protocol, in relation to 10/66 and HCAP family of studies

**Cognitive tests in CADAS**	**Cognitive domains assessed**	**Administered in 10/66**	**Administered in HCAP**
Time, place	Orientation	Similar	Yes
3-word recall: immediate and delayed	Memory	Similar	Yes
Serial 7s	Executive function		Yes
Backward spelling	Executive function		Yes
Object naming, repeating phrase, 3-step task, visual command, sentence writing	Language	Partial (object naming, repeating phrase, 3-step task)	Yes
Overlapping pentagons	Visuospatial	Yes	Yes
10-word list learning; immediate and delayed recall, recognition [22]	Memory	Similar immediate and delayed recall	Yes
Semantic fluency (animal naming)	Language	Yes	Yes
Symbol cancellation [23]	Attention/Speed, Visuospatial		Yes, letter cancelation
Timed Backward counting [24]	Numeracy, Attention/Speed		Yes
Community Screening Instrument for Dementia (CSI-D) [25]	Orientation, language, executive function	Yes	Abbreviated
Partial (immediate): immediate, delayed	Memory	Partial, immediate	Yes
Story recall (robbery): immediate, delayed	Memory		Yes
Telephone Interview for Cognitive Status (TICS): date, place, object naming, naming governor/prime minister [26]	Orientation, language	Yes	Yes
Circle, rhombus, overlapping rectangles, cube drawing: copy, delayed recall [27]	Visuospatial, memory		Yes

#### Informant interview

The Informant interview is another important aspect of the CADAS study protocol. As both 10/66 and HCAP do, CADAS requests that participants identify a key informant: a close family member or friend who knows the participant well, interacts with them frequently so that the family/friend can report on the participants’ activities and function. The CADAS Informant interview includes key elements from 10/66 (e.g. care module, Neuropsychiatric Inventory Questionnaire [28]), HCAP (e.g. Blessed Dementia Rating scale [29], short Informant Questionnaire on Cognitive Decline in the Elderly [30]) as well as the common Community Screening Instrument for Dementia (CSI-D) Cognitive Activities Questionnaire [25]. Table 3 presents the set of informant reports included in CADAS, compared to those administered in 10/66 and HCAP.

**Table 3:** CADAS Informant-Reported Protocol, in relation to 10/66 and HCAP family of studies

**Informant reports in CADAS**	**Domains measured**	**Administered in 10/66**	**Administered in HCAP**
Care module	Care needs, characteristics of caretaker	Abbreviated	Similar
Informant Questionnaire on Cognitive Decline in the Elderly (JORM-IQCODE) [30]	Cognitive and functional damage		Yes
Blessed Dementia Rating Scale [29]	Cognitive and functional damage		Yes
Community Screening Instrument for Dementia (CSI-D) Cognitive Activities Questionnaire [25]	Cognitive activities	Yes	Yes
Zagit Caregiver Burden Interview	Caregiver burden	Yes	
History and Aetiology Schedule Dementia Diagnosis and Subtype (HAS-DDS) [8]	Diagnosis and subtype of dementia	Yes	
Neuropsychiatric Inventory Questionnaire (NPI-Q) [28]	Neuropsychiatric symptoms	Yes	
HRS/HCAP Activities questionnaire	Activities		Yes

#### Blood collection

In the Dominican Republic and Puerto Rico, CADAS will collect blood samples from all consenting participants. Blood samples for the study are collected using a standardized protocol designed to minimize pre-analytical variation and ensure consistency across all sites. Post-collection, the blood samples undergo centrifugation at 1500 to 2000 × g for 10 minutes at 4 °C; red top tubes for serum are centrifuged in the field 20–60 minutes after collection, and EDTA tubes for plasma are centrifuged in a laboratory within 24 hours. Blood samples will be assayed for complete blood count (CBC) and HbA1c levels, the results of which will be returned in a timely manner to the participants. Remaining samples are aliquoted by lab partners of the Universidad*Nacional Pedro Henríquez Ureña *and the University of Puerto Rico, respectively, and stored in cryovials to prevent repeated freeze-thaw cycles. Aliquots will be frozen at −80 degrees Celsius and stored for genetic and biomarker assays (pending future funding). Proposed assays include whole-genome sequencing and Alzheimer’s Disease blood-based biomarkers (Aβ, pTau isoforms, total tau, NfL, GFAP, NRGN, TREM2) to classify participants according to the ATN framework and evaluate genetic associations with biomarker-defined phenotypes.

## Online and Clinical consensus diagnosis

In-person clinician diagnosis of dementia status is not feasible in large population-based samples, thus CADAS is instead training teams of clinicians to provide online clinical consensus dementia diagnoses based on National Institute on Aging and Alzheimer’s Association (NIA-AA) clinical ratings [31] for a subset of CADAS participants (*n*=900). CADAS builds on the online consensus website approaches developed by the Harmonized Diagnostic Assessment of Dementia for the Longitudinal Aging Study in India (LASI-DAD) [32] and Cognition and Dementia in the Health and Aging in Africa Longitudinal Study (HAALSI) [33] studies. In the CADAS online consensus diagnosis, clinicians will assess 1) patients’ cognitive impairment based on cognitive testing; 2) impairment per informant reports; 3) whether impairment interferes with social or occupational functioning; 4) whether it represents a decline; 5) whether it is explained by delirium or psychiatric disorder; and whether it impairs 6) learning and memory, 7) reasoning and complex tasks, 8) visuospatial abilities, 9) language abilities, and/or 10) personality, behavior. In each case, three clinicians provide a first review with domain-specific ratings. Global ratings are generated using the NIA-AA algorithm, and all ratings per case are compared. When ratings differ in computed dementia diagnosis, a team of clinicians is convened to review the case (and others) during an online consensus call. 

In addition, approximately 200 CADAS participants in Cuba will separately have an in-person cognitive evaluation by trained clinicians after the CADAS data collection visit has been completed; the resulting clinical dementia assessment will then be used to validate the dementia assessments made based on CADAS data.

## Data analysis

We will estimate the prevalence of dementia in Puerto Rico, Dominican Republic and Cuba. We will do so using a variety of approaches, including applying the 10/66 dementia classification algorithm [34], attempting to validate an algorithm based on the online consensus diagnoses, and using diagnostic criteria relative to a normative sample [35].

Second, we will investigate life course risk factors for dementia: early-life socioeconomic status and education; midlife risk behaviors and cardiovascular risk factors; and late-life physical, cognitive, social activities, exposome exposures, etc. CADAS is collecting rich data on a broad range of items that may or may not have a direct impact on dementia. These include demographics, age, gender, early-life health and living conditions, work history, social connections, and health behaviors. We plan to estimate the relationship between these factors and cognitive health at older ages and conduct cross-national comparative analyses to better understand the generalizability of these risk factors.

Third, we seek to analyze societal costs of dementia in Puerto Rico, Dominican Republic and Cuba. Specifically, for each country/island, we will characterize the associated costs of dementia in terms of health care utilization, ADL/IADL limitations, co-residence patterns, formal and informal caregiving costs and trade-offs. By using harmonized measures, we can compare the societal costs of dementia among these three Spanish-speaking Caribbean settings, as well as compare with the United States and elsewhere.

## Data Availability

No datasets were generated or analysed during the current study. De-identified data from Puerto Rico and the Dominican Republic will be made publicly available to researchers. Data from Cuba will require an application for use and approval by the principal investigator in Cuba. Research findings will be presented during stakeholder meetings and professional conferences and submitted to peer-reviewed journals.
